# Risk Perception, Preventive Behaviors, and Vaccination Coverage in the Korean Population during the 2009–2010 Pandemic Influenza A (H1N1): Comparison between High-Risk Group and Non–High-Risk Group

**DOI:** 10.1371/journal.pone.0064230

**Published:** 2013-05-17

**Authors:** Jung Yeon Heo, Soung Hoon Chang, Min Jung Go, Young Mee Kim, Sun Hye Gu, Byung Chul Chun

**Affiliations:** 1 Department of Preventive Medicine, Korea University Medical College, Seoul, Korea; 2 Department of Preventive Medicine, Konkuk University, School of Medicine, Chungju, Korea; College of Medicine, Hallym University, Republic of Korea

## Abstract

**Background:**

This study was carried out to estimate the vaccination coverage, public perception, and preventive behaviors against pandemic influenza A (H1N1) and to understand the motivation and barriers to vaccination between high-risk and non–high-risk groups during the outbreak of pandemic influenza A (H1N1).

**Methodology/Principal Findings:**

A cross-sectional nationwide telephone survey of 1,650 community-dwelling Korean adults aged 19 years and older was conducted in the later stage of the 2009–2010 pandemic influenza A (H1N1) outbreak. The questionnaire identified the demographics, vaccination status of participants and all household members, barriers to non-vaccination, perceived threat, and preventive behaviors. In Korea, the overall rate of pandemic influenza vaccination coverage in the surveyed population was 15.5%; vaccination coverage in the high-risk group and non–high-risk group was 47.3% and 8.0%, respectively. In the high-risk group, the most important triggering event for vaccination was receiving a notice from a public health organization. In the non–high-risk group, vaccination was more strongly influenced by previous experience with influenza or mass media campaigns. In both groups, the most common reasons for not receiving vaccination was that their health was sufficient to forgo the vaccination, and lack of time. There was no significant difference in how either group perceived the threat or adopted preventive behavior. The predictive factors for pandemic influenza vaccination were being elderly (age ≥65 years), prior seasonal influenza vaccination, and chronic medical disease.

**Conclusions/Significance:**

With the exception of vaccination coverage, the preventive behaviors of the high-risk group were not different from those of the non–high-risk group during the 2009–2010 pandemic. For future pandemic preparedness planning, it is crucial to reinforce preventive behaviors to avoid illness before vaccination and to increase vaccination coverage in the high-risk group.

## Introduction

Pandemic influenza A (H1N1) arose in North America in April 2009 and subsequently spread worldwide. It caused at least 18,449 casualties in 214 countries by June 7, 2010 [1]. In Korea, the first case of pandemic influenza was confirmed in a 50-year-old woman who had returned from Mexico on May 1, 2009. From mid-August, schools were the epicenters as pandemic influenza A (H1N1) spread widely and quickly into the local community. By the end of October, the influenza-like illness prevalence rate peaked at 45 per 1,000 in the Korean population [2]. During the early stages of the pandemic, a quarantine and isolation policy blocked introduction of the pandemic into the local community. After July, when the epidemic had begun to infiltrate the local community, administration of antiviral agent and a vaccination policy was initiated. Vaccination was the most effective measure for reducing the number of infections, hospitalizations, and deaths. However, it was only available after the pandemic had peaked. The vaccine supply was limited and thus could not be used at a more appropriate time. Therefore, along with the isolation and treatment of the infected, other preventive measures, such as hand washing, mask use, and covering the mouth while coughing, were disseminated through the media to mitigate the damage caused by the pandemic influenza.

During the early phase of the pandemic, a higher mortality rate among young adults compared to that occurring during seasonal influenza was reported. By reporting this statistic widely, the mass media amplified the fear among the general population. The response from the public based on this inaccurate information led to a number of problems, such as a strong demand for unnecessary confirmation testing for pandemic influenza or reluctance to receive the vaccine [3]. Previous studies on the response to the 2002 outbreak of severe acute respiratory syndrome found that people were more likely to comply with health-related recommendations if they believed that the government was providing clear and sufficient information about the outbreak [4], [5]. In addition, the studies suggested that compliance with health-related recommendations such as vaccination and preventive measures would increase if people believed they had a high likelihood of being affected or if they perceived the illness to have a severe consequence.

Those with risk factors such as underlying comorbidity or age exceeding 65 years tend to develop complications from influenza that lead to a high mortality rate [6], [7]. Therefore, we expect that high-risk individuals will be more compliant towards vaccination and preventive behaviors when faced with an influenza pandemic.

This study was conducted to understand the difference in vaccination coverage between high-risk and non–high-risk groups during the 2009–2010 pandemic influenza A (H1N1) in Korea and to analyze the factors influencing the vaccinated and non-vaccinated. In addition, we investigated whether there was a difference between the groups concerning public perception and preventive behavior.

## Materials and Methods

### Cross-sectional telephone survey

This was a nationwide, population-based cross-sectional study. The target study population comprised non-institutionalized individuals aged 19 years or older living in South Korea. Using well-trained professional interviewers, the Hyundae Research Institute®, a professional research company, conducted a telephone survey of 1,650 Korean residents over 19 years of age on 16–26 February 2010. Proportional quota sampling was used to ensure that respondents were demographically representative of the general population, with quotas based on age and region.

Only one participant per household was asked to answer questions about his/her pandemic influenza A (H1N1) vaccination status and that of all family members, perceived threat and preventive behaviors, triggering events for the vaccinated, barriers to vaccination for the non-vaccinated, and sociodemographic factors.

### Questionnaire

Before the interview, the interviewer explained the purpose of the study to all the respondents and obtained verbal informed consent from the respondents who agreed to participate. The questionnaire contained 11 questions Data on demographics, including age, sex, education level, income level, chronic medical disease, and family members according to age group were obtained. Each interview was divided into 2 parts. The first solicited responses concerning the public perception of influenza and preventive behavior, and the second part sought responses concerning pandemic influenza vaccination. Questions in the first part included “How much of a health threat is posed by influenza infection?”, and “What were your preventive measures during the epidemic?” For the second part, the questions included “Did you and your family members receive vaccination against pandemic influenza A (H1N1)?”, “What made you get a vaccination?”, and specifically for the non-vaccinated respondents, “What was the reason for non-vaccination?”

### Definition of high-risk group

In Korea, the priority groups for pandemic influenza vaccination include healthcare workers, children aged 7–18 years, children aged 6 months to 6 years, pregnant women, caregivers for infants aged <6 months, nursing home residents, military personnel, people aged 19–64 years with chronic medical disease, and elderly people aged ≥65 years. Of these groups, special onsite immunization teams visited the 7–18-year-old children at school. This group was involved in mass vaccination. Therefore, it was suspected that the vaccination coverage for this age group was higher compared to other groups who visited medical centers voluntarily. Considering the sample size, people with chronic medical disease and those aged ≥65 years were deemed the high-risk group within the priority groups. Chronic medical diseases included cardiovascular diseases such as congestive heart failure and myocardial infarction, lung disease such as asthma and chronic obstructive pulmonary disease, diabetes, malignancy, chronic liver disease, and rheumatologic diseases such as systemic lupus erythematosus and rheumatoid arthritis.

### Statistical analyses

The response proportions and 95% confidence interval were estimated and compared with the χ^2^ test according to vaccination status and risk group. We used logistic regression analysis to investigate age, sex, education level, and monthly income as potential factors leading to the risk groups deciding to be vaccinated. The difference in the vaccination rates of the risk groups was calculated using the Cochran-Mantel-Haenszel test, controlled by several variables.

### Ethics statement

The study obtained ethics approval from the Institutional Review Board (IRB) of Korea University.

The IRB waived the requirement for written informed consent because the data were analyzed anonymously, but verbal consent was obtained from all respondents before the interview was started.

## Results

### Demographic data

Overall, 1,875 potential respondents were contacted. Of these, 1,650 participated in the telephone survey. The mean age was 44.89±15.18 years and 817 subjects (49.5%) were male. Two hundred and nine subjects (12.7%) were aged ≥65 years and 172 subjects (10.4%) had one or more chronic medical diseases. In total, 313 subjects (19.0%) were classified as high risk, being affected by diabetes (n = 67, 4.1%), cardiovascular disease (n = 66, 4.0%), lung disease (n = 30, 1.8%), malignancy (n = 14, 0.8%), chronic liver disease (n = 12, 0.7%), and rheumatologic disease (n = 8, 0.5%).

### Pandemic influenza vaccination coverage and related factors

The survey established that the pandemic influenza A (H1N1) vaccination coverage was 15.5% among adults aged ≥19 years and 58.4% among elderly people aged ≥65 years; in the high-risk group, vaccination coverage was 47.3% among people with chronic medical conditions and those aged ≥65 years (Table 1). Pandemic influenza vaccination coverage was significantly higher in those at high risk, i.e., people aged ≥60 years and people who had received prior seasonal influenza vaccination. Conversely, there were negative correlations between vaccination coverage and education or income level. Vaccination coverage was 26.3% among all respondents' household members across all age groups (Table 2). Within age groups, the vaccination coverage was highest in children aged 7–12 years, being 84.0%. Generally, school-aged children and adolescents had the highest vaccination rate, followed by children aged ≤6 years (57.2%). The vaccination rate for people aged ≥65 years was 51.5%.

**Table 1:** Rate of pandemic (H1N1) influenza vaccination coverage among study population.

	Vaccinated (%)	Non-vaccinated (%)	Total (%)	*P* value^a^
Total	255 (15.5)	1395 (84.5)	1650	
Sex				0.207
Male	117 (14.3)	700 (85.7)	817 (49.5)	
Female	138 (16.6)	695 (83.4)	833 (50.5)	
Age groups (years)				<0.001
19–29	22 (7.3)	278 (92.7)	300 (18.2)	
30–39	30 (8.5)	325 (91.5)	355 (21.5)	
40–49	33 (8.7)	346 (91.3)	379 (23.0)	
50–59	26 (8.8)	269 (91.2)	295 (17.9)	
60+	144 (44.9)	177 (55.1)	321 (19.4)	
High-risk group	148 (47.3)	165 (52.7)	313 (19.0)	
Age ≥65 years	122 (58.4)	87 (41.6)	209 (12.7)	<0.001
Underlying disease	68 (39.5)	104 (60.5)	172 (10.4)	<0.001
Diabetes	26 (38.8)	41 (61.2)	67 (4.1)	<0.001
Cardiovascular	28 (42.4)	38 (57.6)	66 (4.0)	<0.001
Lung disease	14 (46.7)	16 (53.3)	30 (1.8)	<0.001
Malignancy	5 (35.7)	9 (64.3)	14 (0.8)	0.035^b^
Chronic liver diseases	2 (16.7)	10 (83.3)	12 (0.7)	0.908^b^
Rheumatologic diseases	3 (37.5)	5 (62.5)	8 (0.5)	0.128^b^
Education level^c^				<0.001
Elementary school	74 (46.3)	86 (53.7)	160 (9.9)	
Middle school	27 (19.7)	110 (80.3)	137 (8.5)	
High school	64 (13.3)	416 (86.7)	480 (29.7)	
≥ College/university graduate	81 (9.6)	760 (90.4)	841 (51.9)	
Monthly income^d^ (million Korean won)^e^				<0.001
<1.99	114 (27.6)	299 (72.4)	413 (28.5)	
2.00–3.99	62 (11.1)	499 (88.9)	561 (38.6)	
4.00+	44 (9.2)	434 (90.8)	478 (32.9)	
Seasonal vaccination in 2009–2010 season	174 (34.3)	333 (65.7)	507 (30.7)	<0.001

a χ2 test.

b Fischer's exact test.

c Thirty-two missing values were excluded from the analysis.

d One hundred and ninety-eight missing values were excluded from the analysis.

e Exchange rate based on two million Korean won to US $1,830 and four million Korean won to US $3,660.

**Table 2:** Rate of pandemic influenza (H1N1) vaccination coverage among all household members of the study population.

Age groups (years)	Number of all household members	Number of vaccinated	Vaccination rate (%)	95% CI	
≤6	299	171	57.2	51.6,	62.8
7–12	363	305	84.0	80.3,	87.8
13–15	224	163	72.8	66.9,	78.6
16–18	217	134	61.8	55.3,	68.2
19–64	3,943	413	10.5	9.5,	11.4
≥65	551	284	51.5	47.4,	55.7
Total	5,597	1,470	26.3	25.1,	27.4

95%CI, 95% confidence interval.

### Difference of rate of pandemic influenza vaccination coverage according to risk group

The rate of vaccination coverage of the non–high-risk group was 8.0%, whereas that of the high-risk group was 47.3% (Table 3). The rate of vaccination coverage of the high-risk group was statistically higher even when adjusted according to age, sex, education level, and monthly income (p<0.001).

**Table 3:** Rate of pandemic (H1N1) influenza vaccination coverage among study population according to risk group.

	High risk		Non–high risk		*P* value^a^
	Total	Vaccination (%)	Total	Vaccination (%)	
Sex					<0.001
Male	151	72 (47.7)	666	45 (6.8)	
Female	162	76 (46.9)	671	62 (9.2)	
Age (years)					<0.001
≤59	82	12 (25.6)	1,247	90 (7.2)	
60+	231	127 (55.0)	90	17 (18.9)	
Education level^c^					<0.001
Elementary school	118	64 (54.2)	42	10 (23.8)	
Middle school	53	20 (37.7)	84	7 (8.3)	
High school	65	30 (46.2)	415	43 (8.2)	
≥College/university graduate	74	28 (37.8)	767	53 (6.9)	<0.001
Monthly income^d^ (million Korean won)^e^					
≤1.99	174	90 (51.7)	239	24 (10.0)	
2.00–3.99	60	19 (31.7)	501	43 (8.6)	
4.00+	45	17 (37.8)	433	27 (6.2)	
All	313	148 (47.3)	1337	107 (8.0)	<0.001^b^

a Cochrane-Mantel-Haenzel test.

b Adjusted by age, sex, education level, and monthly income with logistic regression analysis.

c Thirty-two missing values were excluded from the analysis.

d One hundred and ninety-eight missing values were excluded from the analysis.

e Exchange rate based on two million Korean won to US $1,830 and four million Korean won to US $3,660.

### Triggering events and barriers to pandemic influenza vaccination by risk group

**Figure 1:**
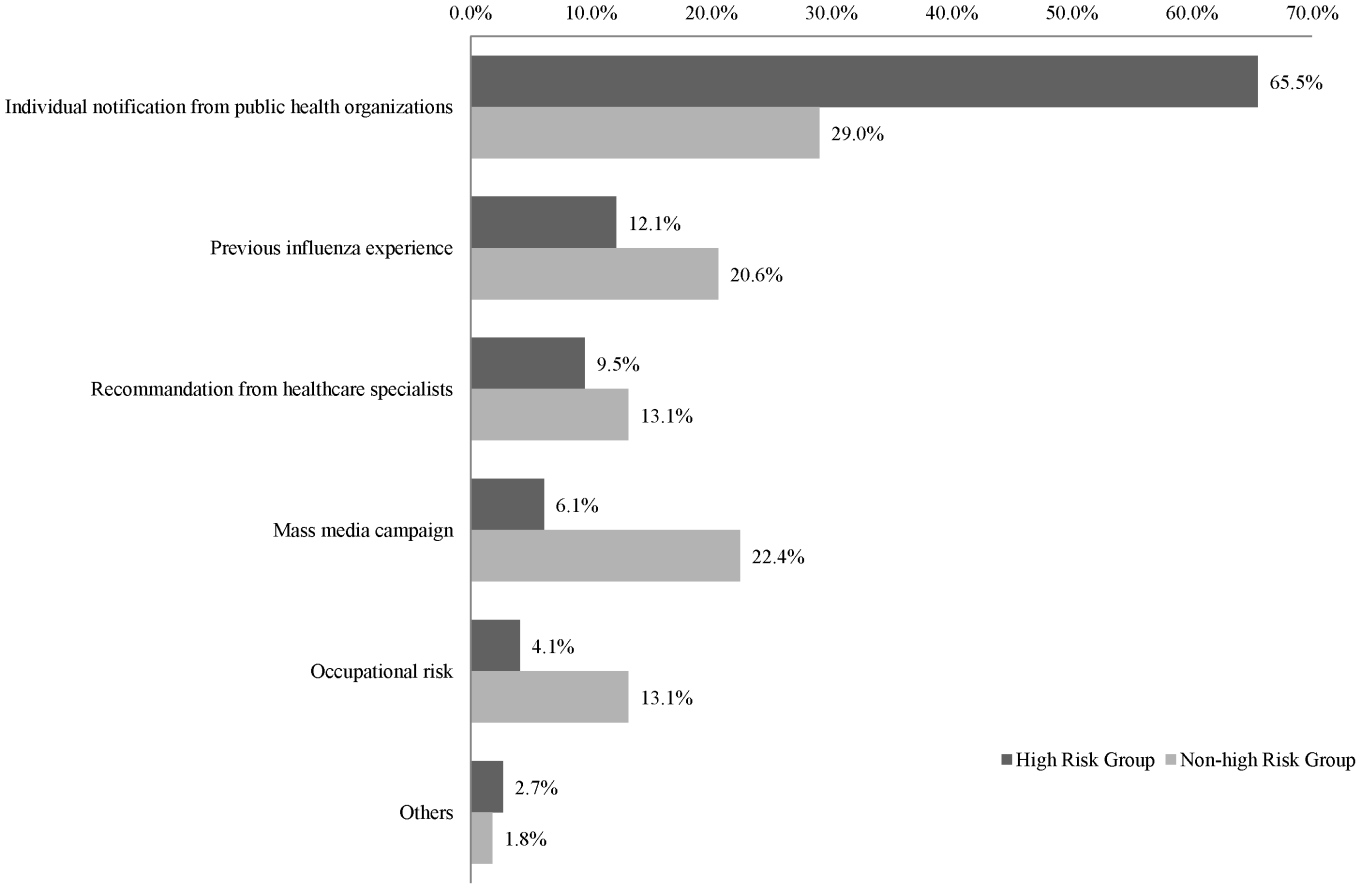
Triggering events for pandemic influenza A (H1N1) vaccination according to risk group.

The triggering events between the high-risk and non–high-risk groups are compared in Figure 1. For both groups, the most important triggering event was receiving a notice from a public health organization. In particular, two-thirds of the vaccinations in the high-risk group were triggered in this manner (65.5%), followed by previous experience from seasonal influenza (12.1%), recommendation from healthcare specialists (9.5%), and mass media campaigns (6.1%). In the non–high-risk group, apart from notices from public health organizations, mass media campaigns (22.4%) and previous experience from seasonal influenza (20.6%) were important triggering events.

**Figure 2:**
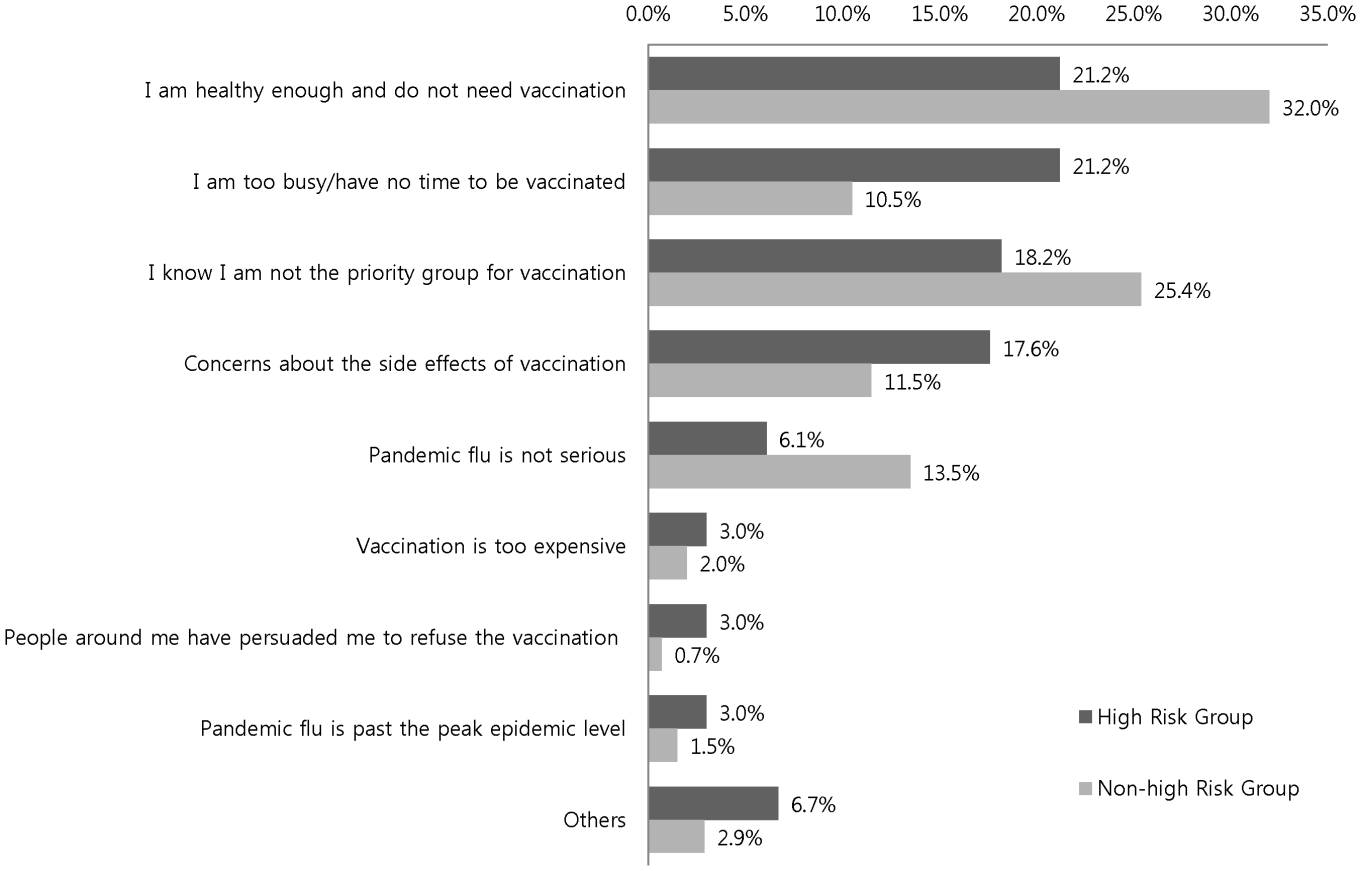
Barriers to pandemic influenza A (H1N1) vaccination according to risk group.

Among the non-vaccinated, the reasons for not being vaccinated between the high-risk and non–high-risk groups are compared in Figure 2. Within the high-risk group, the major reasons for avoiding vaccination was the belief in sufficiently robust personal health (21.2%), lack of time (21.2%), being unaware of their being in a priority group (18.2%), and concern over vaccine side effects (17.6%). More than half of the respondents in the non–high-risk group believed they were healthy enough not to require vaccination (32.0%) or that they were not in a priority group (25.4%).

### Perception and preventive behaviors for pandemic influenza according to risk group

**Figure 3:**
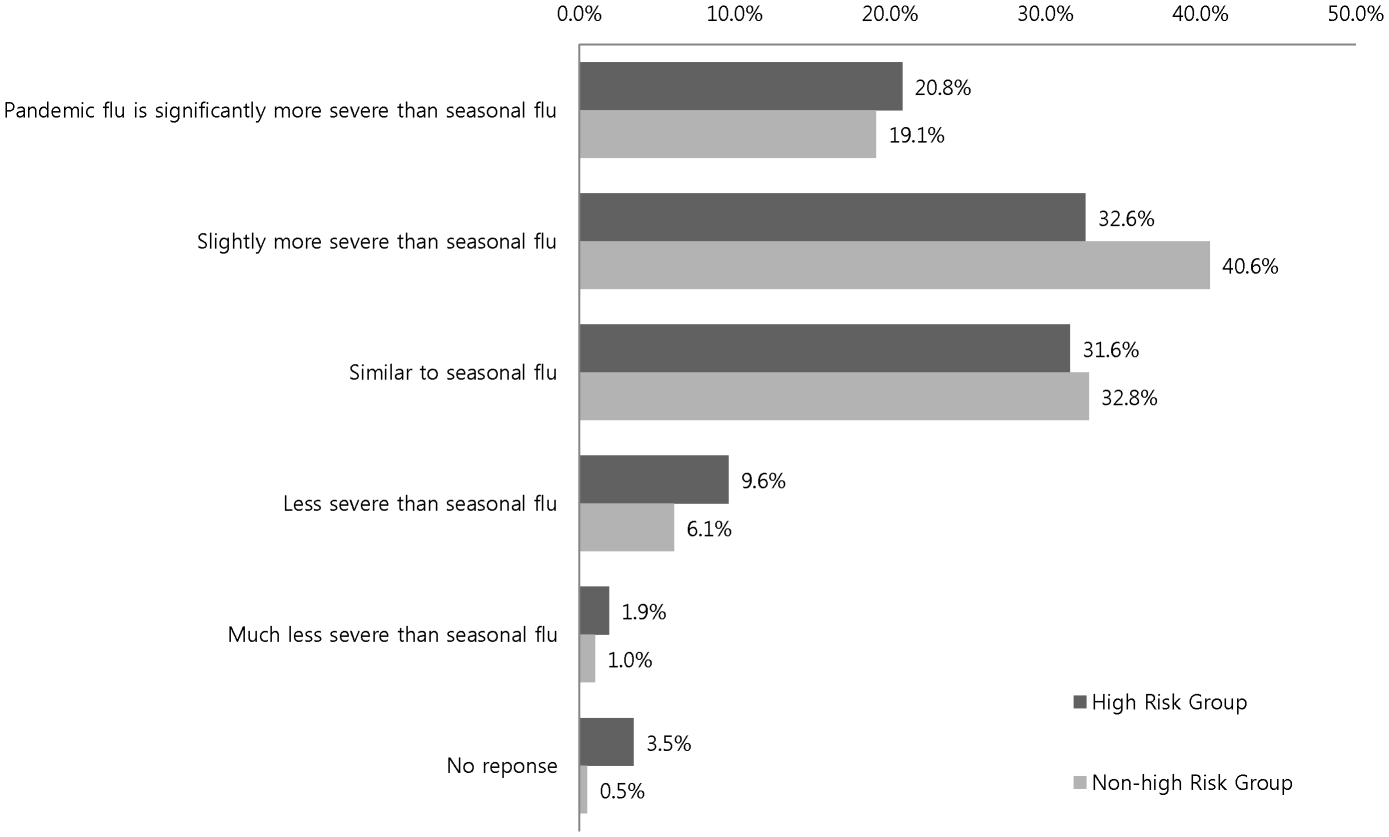
Perception of pandemic influenza A (H1N1) severity according to risk group (p = 0.392 by χ2 test).

Figure 3 displays data concerning how the respondents perceived the severity of the pandemic influenza compared to seasonal influenza. Both the high-risk and non–high-risk groups believed that the pandemic influenza was only slightly more severe than seasonal influenza (32.6% vs. 40.6%, respectively) or similarly severe (31.6% vs. 32.8%, respectively). In addition, about one-fifth of each group believed that the pandemic influenza was significantly more severe than seasonal influenza (high risk vs. non–high risk, 20.8% vs. 19.1%, respectively). However, there was no significant difference between the groups regarding the perceived threat of pandemic influenza (*p = *0.392).

**Figure 4:**
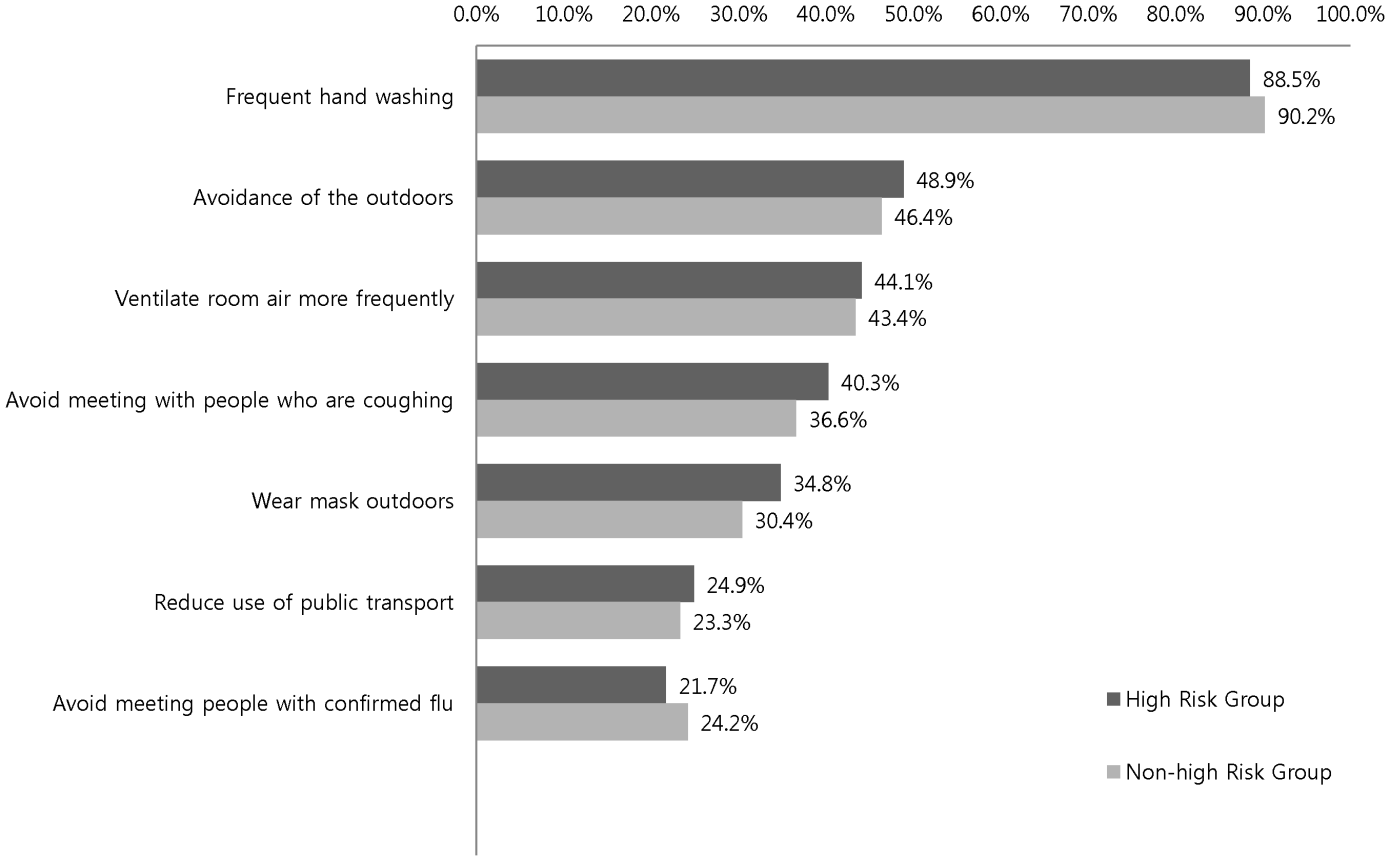
Preventive behaviors during pandemic influenza A (H1N1) according to risk group (p = 0.307 by χ2 test).

Figure 4 depicts data concerning preventive behaviors adopted during pandemic influenza. The most common preventive behavior in the high-risk and non–high-risk groups was frequent hand washing (88.5% vs. 90.2%, respectively), followed by avoidance of the outdoors (48.9% vs. 46.4%, respectively), frequent use of ventilation (44.1% vs. 43.4%, respectively), and avoidance of those who were coughing (40.3% vs. 36.6%, respectively). There was no significant difference in the preventive behaviors in both groups (*p = *0.307).

### Likelihood of vaccination

Table 4 displays the results from the multiple logistic regression analysis of the factors involved in pandemic influenza vaccination. The most significant factor influencing vaccination against pandemic influenza A (H1N1) was age ≥65 years (odds ratio [OR] 5.84). Other factors such as prior seasonal influenza vaccination (OR 3.68) and chronic medical disease (OR 2.08) were also statistically significant.

**Table 4:** Multiple logistic regression analysis on factors influencing pandemic (H1N1) influenza vaccination.

	OR	95% CI	*P* value
Female	1.02	0.72–1.45	0.923
Age ≥65 years	5.84	3.59–9.49	<0.001
Underlying disease	2.08	1.31–3.12	0.002
Education level			
Elementary school	1.19	0.43–1.65	0.838
Middle school	1.08	0.58–2.11	0.763
High school	1.21	0.54–1.16	0.826
≥ College/university graduate	1.00		
Monthly income (million Korean won)^a^			
≤1.99	1.14	0.52–1.57	0.641
2.00–3.99	1.01	0.64–1.56	0.988
4.00+	1.00		
Severity perception			
More serious than seasonal flu	1.07	0.49–1.76	0.826
Similar to seasonal flu	1.11	0.46–1.76	0.757
Less serious than seasonal flu	1.00		
Prior seasonal flu vaccination	3.68	2.55–5.29	<0.001

a Exchange rate based on two million Korean won to US $1,830 and four million Korean won to US $3,660.

OR, odds ratio; 95% CI, 95% confidence interval.

## Discussion

In this study, the cross-sectional telephone survey conducted determined that self-reported pandemic influenza vaccination coverage in adults aged >19 years was 15.5%. The vaccination coverage of all respondents' household members across all age groups was 26.3%. This finding was consistent with the 26.1% recorded in the Korean Immunization Registry (KIR) study [8]. However, the KIR study found that vaccination coverage in the elderly (age ≥65 years) was 38.0%, which was lower than the findings of our telephone survey. The KIR information is highly reliable as it used vaccination uptake data. By contrast, the telephone survey confirmed vaccination coverage through self-reported data. Therefore, it is possible that vaccination coverage was overestimated in those aged ≥65 years. Yet, although selection bias might apply in the severely ill and in the elderly, previous studies support the validity of self-reported vaccination coverage [9]–[11]. Moreover, the KIR only provides information on age, sex, and the priority group type of the vaccinated. On the other hand, our survey data provide a range of information, such as the triggering events for the vaccinated or the barriers encountered by the non-vaccinated; the perception concerning pandemic influenza can be a clear advantage. Therefore, the data from this survey address not only pandemic influenza vaccination coverage, but can distinguish differences between factors relevant to pandemic influenza vaccination or non-vaccination, public perception, and preventive behaviors against pandemic influenza between high-risk and non–high-risk groups.

In univariate analysis, age ≥60 years, chronic medical disease, low education, and low income were associated with a high tendency for vaccination. The findings are consistent with the results of a seasonal influenza vaccination study in Korea [12]. Furthermore, when age, sex, education, and income were adjusted, the difference in vaccination coverage between the high-risk group and the non–high-risk group was statistically significant. The finding showed that in pandemic influenza vaccination, vaccination campaigns targeted towards those at high risk were partially successful. In Korea, there are four aspects to targeting vaccination towards the high-risk group. First, people registered as having a chronic medical disease in the Health Insurance Review Agency database received vaccination information in the mail. Second, the elderly (i.e., those aged ≥65 years), those known to be financially vulnerable, and nursing home residents received free vaccinations at public health centers. Third, pregnant women and people with chronic medical diseases were vaccinated at private clinics at the overall cost of 15,000 Korean won (US $14). Fourth, an information dissemination campaign was conducted through the public media. However, despite these steps, pandemic influenza vaccination coverage in the high-risk group was lower when compared to seasonal influenza vaccination. In Korea, the high mortality rate in people aged ≥60 years and with chronic medical disease during the pandemic influenza A (H1N1) indicates that a more aggressive vaccination program is needed for the high risk group [13].

In the high-risk group, the major triggering event for pandemic influenza vaccination was receiving a notice from a public health organization, rather than the more easily accessible mass media campaigns. The respondents' major reasons for pandemic influenza non-vaccination were “confidence in health” and “not enough time to get vaccinated.” These reasons for non-vaccination were consistent with a previous study carried out in Korea [12]. Studies in France, Australia, the United States, and Hong Kong demonstrated that vaccine safety and side effects were the most significant barriers to vaccination, whereas fear of vaccination side effects was relatively low in Korea [14]–[17]. This could indicate a cultural difference in the perception towards vaccination. Factors associated with pandemic influenza vaccination are important clues for increasing vaccination uptake. Aside from the high-risk group, the non–high-risk group considered receiving a notice from a public health organization the most important triggering event. A study conducted in the US reported that people who received information on the safety and efficacy of a vaccine from public health organizations or healthcare providers rather than media, or family or friends had a higher probability of being vaccinated [18]. This strongly indicates that public health organizations should publicize vaccination more actively. In particular, it is necessary to provide information about influenza and vaccine efficacy to those at high risk to motivate them to receive vaccination.

More than half of the respondents perceived pandemic influenza as being more serious than seasonal influenza, and about 20% perceived pandemic influenza as a very severe disease. However, perception of the severity of pandemic influenza was not the influencing factor for vaccination uptake. This result was different from studies in the United Kingdom, Greece, and Australia, where a positive correlation was reported between perception of the severity of pandemic influenza and compliance with vaccination [19]–[21]. Liao et al. performed a longitudinal study during a pandemic influenza period, and reported that among those who intended to receive a vaccination, only 10% actually were vaccinated [22]. Empirical studies on intention–behavior relation have shown that intention had a moderate effect on action [23], [24]. In Korea, the mass media reported the adverse effects and safety issues of the adjuvant influenza vaccine during the pandemic influenza outbreak, which may have fueled a negative perception of vaccination.

As a response to the threat of pandemic influenza, more than 90% of people will adopt one or more preventive behaviors. However, apart from hand washing, less than 50% of the respondents practiced preventive behaviors such as avoiding crowded places or public transport. In addition, there was no significant difference in preventive behaviors between the high-risk and non–high-risk groups. Early use of a vaccine is not applicable during pandemic influenza, thus preventive behaviors are crucial to mitigate further spread of the infection. Therefore, efforts to enhance the knowledge of preventive behaviors could be the main strategy in preparing for future pandemic influenza.

This study had several limitations. First, the high-risk group consisted of the elderly (aged >65 years) and people with chronic medical conditions. Therefore, other priority groups such as pregnant women and healthcare workers were not included. Second, chronic medical conditions and vaccination status relied entirely on self-reporting. The vaccination rate was similar when compared with KIR data, but the rate was relatively overestimated in chronic medical conditions. Third, this was an observational study, which is inherently limited for explaining correlations.

Despite these limitations, this study does suggest that disseminating accurate information about influenza and the necessity of vaccination by the government or public health organizations appears to be a valuable component for those at high risk.

In summary, both perceived threat and preventive behaviors of the high risk group were not markedly different from those of the non-high risk group during the 2009 pandemic in Korea, except for vaccination coverage. Triggering events for vaccination was the major difference between high risk and non-high risk groups. Notice from public health organization was the most important event for triggering the decision to getting vaccination in those at high risk. It is also important to inform those at high risk to practice preventive behaviors to avoid getting ill before vaccination.
